# The Efficacy and Safety of Different Kinds of Laparoscopic Cholecystectomy: A Network Meta Analysis of 43 Randomized Controlled Trials

**DOI:** 10.1371/journal.pone.0090313

**Published:** 2014-02-28

**Authors:** Lun Li, Jinhui Tian, Hongliang Tian, Rao Sun, Quan Wang, Kehu Yang

**Affiliations:** 1 The First Clinical College, Lanzhou University, Lanzhou, China; 2 Evidence Based Medicine Center, School of Basic Medical Sciences, Lanzhou University, Lanzhou, China; University of Nebraska Medical Center, United States of America

## Abstract

**Background and Objective:**

We conducted a network meta analysis (NMA) to compare different kinds of laparoscopic cholecystectomy [LC] (single port [SPLC], two ports [2PLC], three ports [3PLC], and four ports laparoscopic cholecystectomy [4PLC], and four ports mini-laparoscopic cholecystectomy [mini-4PLC]).

**Methods:**

PubMed, the Cochrane library, EMBASE, and ISI Web of Knowledge were searched to find randomized controlled trials [RCTs]. Direct pair-wise meta analysis (DMA), indirect treatment comparison meta analysis (ITC) and NMA were conducted to compare different kinds of LC.

**Results:**

We included 43 RCTs. The risk of bias of included studies was high. DMA showed that SPLC was associated with more postoperative complications, longer operative time, and higher cosmetic score than 4PLC, longer operative time and higher cosmetic score than 3PLC, more postoperative complications than mini-4PLC. Mini-4PLC was associated with longer operative time than 4PLC. ITC showed that 3PLC was associated with shorter operative time than mini-4PLC, and lower postoperative pain level than 2PLC. 2PLC was associated with fewer postoperative complications and longer hospital stay than SPLC. NMA showed that SPLC was associated with more postoperative complications than mini-4PLC, and longer operative time than 4PLC.

**Conclusion:**

The rank probability plot suggested 4PLC might be the worst due to the highest level of postoperative pain, longest hospital stay, and lowest level of cosmetic score. The best one might be mini-4PLC because of highest level of cosmetic score, and fewest postoperative complications, or SPLC because of lowest level of postoperative pain and shortest hospital stay. But more studies are needed to determine which will be better between mini-4PLC and SPLC.

## Background

Laparoscopic cholecystectomy (LC) has been considered the golden standard for cholecystectomy to manage benign gallbladder disease since 1986 [1]-[3]. Usually, the standard LC is done using four trocars [3]. These include one port for the camera; one port for instruments used to carry out the dissection, diathermy, clip application; and two ports for manipulation of the gallbladder for adequate exposure of the field of surgery [4]. The fourth (lateral) trocar is used to grasp the fundus of the gallbladder so as to expose Calot’s triangle [3], [5]. With increasing surgeon experience, it was argued that the fourth trocar may not be necessary, and LC can be performed safely without using it [3], [5]. As a result, three ports laparoscopic cholecystectomy (3PLC) was developed [6], [7]. It was thought that reduced size, smaller incision, and fewer ports for LC will improve cosmetic results, decrease pain, and minimize postoperative complication [8], [9]. So a trend toward even more minimally invasive approaches, such as smaller ports, mini-ports, and reduced ports, has led to the advent of laparoscopic surgery and its continuous development of laparoscopic surgery [10]. Until 1997, Navarra et al. [11] described the first single port laparoscopic cholecystectomy (SPLC), the LC underwent four stages: four ports (4PLC), three ports (3PLC), two ports (2PLC) and single port (SPLC) according to reduced ports. Then a mini-laparoscopic cholecystectomy (mini-PLC) with smaller ports and incisions was also developed. It was said that SPLC represents the next step in laparoscopic surgery in further reducing the invasiveness of surgical procedures with cosmetic advantages [12]. Although current guidelines recommend performing cholecystectomy via laparoscopy [13], we were not sure what kinds of LC will be the golden standard with minimizing morbidity, decreasing pain and improving cosmetic results. So we conducted a network meta analysis [NMA] to compare different kinds of LC (SPLC, 2PLC, 3PLC, 4PLC, and four ports mini-laparoscopic cholecystectomy (mini-4PLC)).

## Methods

We did this systematic review of the available literature in accordance with the PRISMA guidelines [14] for the conduct of meta-analyses of intervention trials.

### Data sources

PubMed, the Cochrane library, EMBASE, and ISI Web of Knowledge were searched to find randomized controlled trials (RCTs) and meta analysis using laparoscopic cholecystectomy. Medical Subject Headings terms were also added in all searches for Pubmed, Embase, and the Cochrane Library. Reference lists from the meta-analysis, review articles about this topic and identified trials were hand-searched to identify further relevant citations. The search strategy was developed by two reviewers (Lun Li and Jinhui Tian who is a professional searcher over ten years’ experience) and peer-reviewed by a third reviewer (Kehu Yang). And the searches were conducted independently by two reviewers (Lun Li and Jinhui Tian) using the same search strategy to avoid the potential mistakes by anyone of them. The search was conducted in August 2013 without language, date, and publication status restrictions; differences were checked by each other and resolved by discussion. The search was updated in 2013, 1^ST^ December.

### Inclusion criteria and study selection

The study type should be RCT which used randomized methods according to what they reported. Those studies which used quasi-randomized methods according to what they reported were excluded. The studies should compare two or three surgery instruments (SPLC, 2PLC, 3PLC, 4PLC, and mini-4PLC). SPLC was defined as laparoscopic excision of the gallbladder performed through a single abdominal incision using either a multiport device or different individual ports through the same single skin incision [15]. For 2PLC, 3PLC, and 4PLC, the instruments should be at least 5 mm. For mini-4PLC, two to three of the four instruments should be at least less than 5 mm. Only published articles in English were included, meeting abstracts, and unpublished data were not included in this NMA.

Two independent reviewers (Lun Li and Hongliang Tian) selected the retrieved citations based on titles and abstracts, and full-texts of potential eligible studies were read to decide whether to include based on inclusion criteria. Disagreements were resolved by discussion, and if not, a third reviewer (Kehu Yang) was involved.

### Data abstraction and quality assessment

Data was entered into an Excel database by two authors (Lun Li and Jinhui Tian). The following fields were abstracted: country, patient characteristics (age, sex and other baseline characteristics), disease, follow-up duration, and outcomes. Outcomes were extracted preferentially by intention to treat method. Any disagreements were resolved by a third reviewer (Kehu Yang).

The methodological quality was evaluated by two independent reviewers (Lun Li and Rao Sun) and resolved differences by consultation with a third reviewer (Kehu Yang). The following items were assessed according Cochrane handbook 5.0 [16]: randomization, blinding, concealed allocation, selective reporting, incomplete outcome data, and other biases.

### Data analysis

The outcomes we evaluated were postoperative pain using visual analogue scale (VAS) at the first day, the number of patients who needed additional analgesics, postoperative complications, intra-operative blood loss, cosmetic score, hospital stay and operative time.

Direct pair-wise meta analysis (DMA) was conducted by Review Manager Version 5.0. For dichotomous outcomes, results were expressed as odds ratio (OR) with 95% confidence interval (CI). If there were continuous scales of measurement, the mean difference (MD) was used to assess the effects of treatment. The percentage of variability across trials attributable to heterogeneity beyond chance was estimated with the I^2^ statistic, which was deemed significant when p was less than 0.05 or I-square was more than 50%. Data was pooled using the fixed-effect model but the random-effects model was also considered to ensure robustness of the model in case of significant heterogeneity.

When direct evidence was lacked, indirect treatment comparison meta analysis (ITC) was retrieved from available evidence. Indirect data was got using ITC software (http://www.cadth.ca/en/resources/about-this-guide/download-software). Here we only calculated an indirect result between two comparisons. For example, if there were two comparisons (A vs. B, B vs. C), an indirect result (A vs. C) was calculated. If there were three or more comparisons (A vs. B, B vs. D, D vs. C), we did not carry an indirect calculation, although it is feasible. For those with different pathways to produce the indirect evidence (we mean different comparators, such as A vs. B, B vs. C and A vs. D, D vs. C), we calculated different indirect results and then the pooled indirect results were calculated using inverse variance method and each estimate is ‘weighted’ by the inverse of the variance.

Network Meta-Analysis (NMA) is a technique to meta-analyze more than two drugs at the same time. Using a full Bayesian evidence network, all indirect comparisons are taken into account to arrive at a single, integrated, estimate of the effect of all included treatments based on all included studies. NMA was conducted using ADDIS software. We also produced the rank probability plot by ADDIS software to show which LC was the best. The data was expressed as odds ratio (OR) or MD with 95% Credibility Interval (CrI).

For inconsistency, we undertook a node-splitting analysis by ADDIS software to assess whether direct and indirect evidence on the split node is in agreement [17]. Meanwhile, the methods described by Song [18] were also used to test the difference between DMA or ITC and NMA evidence. A Z value was calculated to show the difference. If the absolute value of Z was more than 1.645, we thought the p value for Z test was less than 0.05. It is deemed significant when p was less than 0.05.

## Results

### Search results

**Figure 1:**
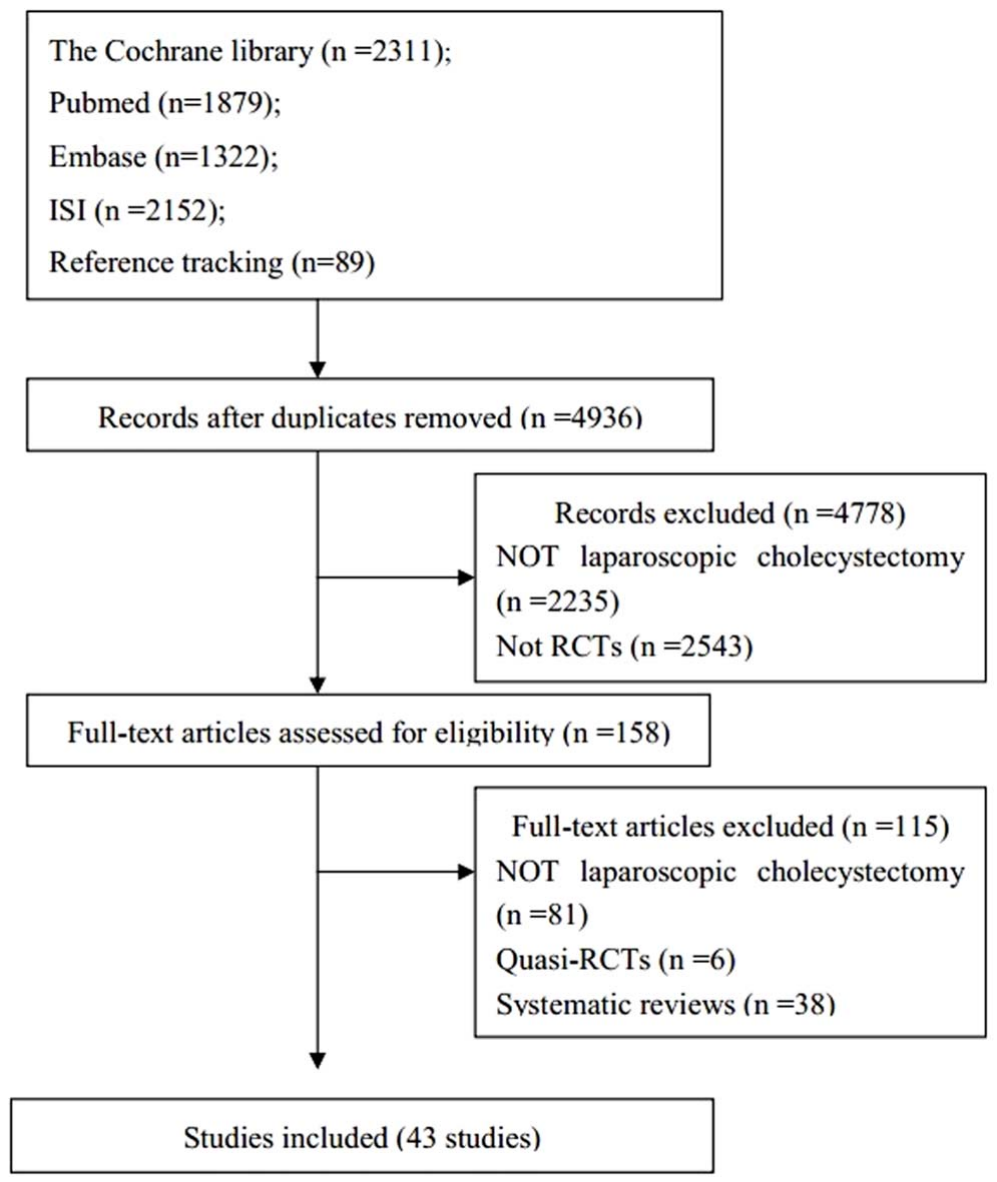
The flow chart.

We got 7644 citations from databases and 89 citations from reference checking. Finally we included 43 RCTs [5]–[7], [19]–[58]. The searching results and selection process was presented in Figure 1.

### Characteristics of included studies

Six studies [20], [27], [44], [46], [56], [58] compared SPLC with 3PLC, two studies [24], [45] compared 2PLC with 4PLC, five studies [5]–[7], [28], [58] compared 3PLC with 4PLC, 18 studies [25], [26], [29], [34], [35], [37]–[41], [43], [47], [49], [52]–[55], [58] compared SPLC with 4PLC, 15 studies [19], [21]–[23], [30]–[33], [36], [42], [47], [48], [50], [51], [57] compared 4PLC with mini-4PLC. LC in all included studies was elective, although some studies included patients with acute cholecystitis. And other characteristics of included studies were presented in table 1.

**Table 1:** Characteristics of included studies.

Study	Country	I	C	Sample size		Gender I:C (F/M)	Age (I:C)	BMI (I:C)
				I	C			
Aprea 2011	Italy	SPLC	3PLC	25	25	13/12:19:6	45.5±9.4: 44.0±10.0	25.9±5.8:23.7±4.6
Cao 2011	China	SPLC	3PLC	57	51	34/23:29/22	62.2±5.1:59.7±4.4	28.6±4.4:29.1±5.1
Pan 2013	China	SPLC	3PLC	49	53	26/23:31:22	43.8±14.0:45.2±11.0	24.3±6.0:25.1±5.0
Rasić 2010	Croatia	SPLC	3PLC	48	50	26/22:32/18	44±6:44±5.7	27±4:27±4
Zheng 2012	China	SPLC	3PLC	30	30	17/13:14:16	43.6±11.3:46.8±14.4	24.7±3.4:25.9±4.1
Bresadola 1999	Italy	2PLC	4PLC	28	37	19/9:22/15	42±20:45±15	-
Poon 2003	China	2PLC	4PLC	58	57	33/25:29/28	52.3±14.9:53.4±13.1	-
Cerci 2007	Turkey	3PLC	4PLC	73	73	54/19:55/18	50.08±12.5:49.77±13.6	29.2:28.7
Kumar 2007	Nepal	3PLC	4PLC	36	39	30/6 :32/7	38.22±13.67:39.13 ±14.10	-
Trichak 2003	Thailand	3PLC	4PLC	100	100	75/25:73:27	53.22±15.31:53.74±15.05	-
Gupta 2005	India	3PLC	4PLC	40	40	-	-	-
Brown 2013	USA	SPLC	4PLC	40	39	29/11:32/7	42:43	29.4±5.1:30.3±6.9
Bucher 2011	Switzerland	SPLC	4PLC	75	75	-	42:44	26:25
Chang 2013	Singapore	SPLC	4PLC	24	26	14/10:16:10	49.46±11.39:51.15±12.31	24.13±4.21:27.65±7.79
Lai 2011	China	SPLC	4PLC	24	27	16/8:16:11	51.7±13.3:54.3±12.0	25±3.0:24.4±2.8
Lirici 2010	Italy	SPLC	4PLC	20	20	14/6:14/6	45:50	25:27
Luna 2013	Brazil	SPLC	4PLC	20	20	-	-	-
Ma 2011	Portland	SPLC	4PLC	21	22	-	57.3±16:45.8±11.9	28.2±5.3:30.7±6.1
Madureira 2013	Brazil	SPLC	4PLC	28	29		50:56	27.5:25
Marks 2013	USA	SPLC	4PLC	119	81	91/28:57/24	45.8:44.0	29:30.9
Mehmood 2010	Pakistan	SPLC	4PLC	30	30	28/2:26:4	44.42±8.59:42.67±9.05	-
Ostlie 2013	USA	SPLC	4PLC	30	30	24/6:24/6	14.0±3.2:13.3±3.3	-
Saad 2013	Germany	SPLC	4PLC/ mini-4PLC	35	35/35	28/7:29/6:29/6	45:49:44	25.4:25.4:25.3
Sasaki 2012	Japan	SPLC	4PLC	27	27	14/13:14/13	56.6±14.2:58.2±12.3	-
Sinan 2012	Turkey	SPLC	4PLC	17	17	13/4:9/8	48.5±8.9:48.7±4.3	27.3±3.1:27.2±2.9
Tsimoyiannis 2010	Greece	SPLC	4PLC	20	20	15/5:19:1	49.2±16.9:47.9±9.8	-
Yilmaz 2013	Turkey	SPLC	4PLC	43	40	34/9:27/13	48.5±12.0:51.0±9.0	24.2±4.0:23.3±3.0
Zapf 2013	USA	SPLC	4PLC	49	51	42/7:34:17	44.2±16.2:50.9±18.2	29.1±6.5:30.0±6.3
Alponat 2002	Turkey	mini-4PLC	4PLC	17	22	15/2:18:4	45.8±13.3:49.7±11.8	26.8±4.3: 25.7±4.9
Bignell 2013	UK	mini-4PLC	4PLC	40	40	29/11:36:4	54:52	-
Bisgaard 2000	Denmark	mini-4PLC	4PLC	13	13	13/3:9:4	46:53	25:26
Bisgaard 2002	Denmark	mini-4PLC	4PLC	25	27	22/3:20:7	47:48	26:27
Cheah 2001	Singapore	mini-4PLC	4PLC	37	38	23/14:21/17	49:52	-
Decarvalho 2013	Belgium	mini-4PLC	4PLC	18	23	16/2:18:5	47±14:52±19	24.6±3.3:24.3±3.5
Hsieh 2003	China	mini-4PLC	4PLC	35	29	19/15:15/14	55.7±17.7:54.5±17.6	-
Huang 2003	China	mini-4PLC	4PLC	54	30	37/33:18/12	49.05±1.84:48.2±14.7	24.3±4.48:24.3±5
Look 2001	Singapore	mini-4PLC	4PLC	28	36	16/12:21/15	53.4±12.3:51.3±14.4	-
Novitsky 2005	USA	mini-4PLC	4PLC	34	33	29/4:26/8	46.7±12.1:41.8±12.4	-
Sarli 2003	Italy	mini-4PLC	4PLC	67	68	37/29:34/34	53:53	27.3:26.2
Schmidt 2002	Germany	mini-4PLC	4PLC	20	20	-	52.4±15.5/54.07±11.9	-
Schwenk 2000	Germany	mini-4PLC	4PLC	25	25	18/7:17/8	44:52	21.7:22.9
Ainslie 2003	UK	mini-4PLC	4PLC	21	19	-	58:49	24.5:27.7
Khorgami 2013	Iran	SPLC	3PLC/4PLC	30	30/30	22/8:20/10:21:9	43.81±12.7:41.7±11.2:41.5±11.1	27.9±4.3:28.6±4.5:26.7±4

I: intervention group; C: control group; F: female; M: male.

### Quality assessment results

All studies mentioned randomization, but only 13 studies reported the details of the randomized methods and 17 studies mentioned the details of concealed allocations. 11 studies mentioned the methods of blinding, however, patients and assessors were blinded in five studies, patients were blinded in three studies, assessors were blinded in two studies and surgeons were blinded in one study. (Table 2).

**Table 2:** Quality assessment results.

Study	Randomization	Allocation concealed	Blinding	Incomplete data	Selective reporting	Other bias
Aprea 2011	M	Y	N	N	U	U
Cao 2011	M	Y	D	N	U	U
Pan 2013	Y	Y	U	U	U	U
Rasić 2010	Y	U	U	N	N	U
Zheng 2012	Y	Y	U	Y	N	U
Bresadola 1999	M	U	U	N	N	U
Poon 2003	M	U	M	U	U	U
Cerci 2007	M	U	U	U	U	U
Kumar 2007	M	U	U	U	U	U
Trichak 2003	M	U	U	U	U	U
Gupta 2005	M	U	U	U	U	U
Brown 2013	M	U	P	U	U	U
Bucher 2011	Y	U	U	N	N	U
Chang 2013	M	U	D, P	Y	N	U
Lai 2011	Y	Y	U	N	N	U
Lirici 2010	M	Y	P	N	U	U
Luna 2013	M	U	U	U	U	U
Ma 2011	M	U	U	U	N	U
Madureira 2013	M	U	U	Y	N	U
Marks 2013	M	U	U	U	Y	U
Mehmood 2010	M	Y	U	U	N	U
Ostlie 2013	Y	U	U	U	U	U
Saad 2013	Y	Y	D, P	N	N	U
Sasaki 2012	Y	U	U	Y	N	U
Sinan 2012	Y	U	U	Y	U	U
Tsimoyiannis 2010	M	Y	U	N	N	U
Yilmaz 2013	M	U	U	U	U	U
Zapf 2013	Y	U	U	U	U	U
Alponat 2002	M	U	U	U	U	U
Bignell 2013	M	U	U	U	U	U
Bisgaard 2000	M	Y	D, P	Y	N	U
Bisgaard 2002	Y	Y	U	Y	Y	Y
Cheah 2001	M	Y	U	U	U	U
Decarvalho 2013	M	Y	U	N	N	U
Hsieh 2003	M	U	U	Y	Y	U
Huang 2003	M	Y	U	Y	N	U
Look 2001	M	U	P	U	U	U
Nvitsky 2005	Y	U	D, P	Y	Y	U
Sarli 2003	M	Y	D	Y	N	U
Schmidt 2002	M	U	S	N	Y	U
Schwenk 2000	M	U	U	U	Y	U
Ainslie 2003	M	U	U	Y	N	U
Khorgami 2013	Y	Y	D, P	N	N	U

M: mentioned; U: unclear; N: no; D: blinded to data collectors; P: blinded to patient; S: blinded to surgeon; Y: yes, adequately reported.

### Direct pair-wise meta analysis (DMA)

According to the results of DMA, we could see that SPLC was associated with more postoperative complications and higher cosmetic score than 4PLC, longer operative time and higher cosmetic score than 3PLC, more postoperative complications than mini-4PLC. Mini-4PLC was associated with longer operative time than 4PLC. No significantly statistical differences were found in other outcomes between different comparisons. (Table 3, Table 4, Table 5).

**Table 3:** Meta analysis for postoperative pain, additional analgesics and intra-operative blood loss.

	Postoperative pain		Pain control		Blood loss	
	DMA/ITC^#^	NMA^&^	DMA/ITC^$^	NMA@	DMA/ITC^#^	NMA^&^
mini-4PLC-4PLC	–0.30 [–1.38, 0.78]d	–0.32 (–1.40, 0.77)	1.00 [0.38, 2.64]d	0.83 (0.30, 2.06)	–6.37 [–26.97, 14.23]d	–8.07 (–27.26, 12.67)
mini-4PLC-3PLC	0.29 [–0.86 1.44]i	0.30 (–1.22, 1.92)	*0.90 [0.31, 2.59]i(p)	0.79 (0.12, 3.86)	–5.65 (–26.62 15.32)i	–7.21 (–27.14, 13.81)
mini-4PLC-2PLC	–0.29(–1.40 0.82)i	–0.13 (–2.48, 2.26)				
mini-4PLC-SPLC	0.42(–1.04 1.88)i	0.38 (–0.93, 1.73)	0.84 [0.27, 2.65]d	1.51 (0.39, 4.88)	–6.22 (–26.98 14.54)i	–7.83 (–27.04, 12.78)
4PLC -3PLC	0.59 [0.20, 0.98]d	0.63 (–0.48, 1.73)	1.61 [0.41, 6.67]d	0.95 (0.20, 3.99)	0.72 (–3.2 4.64)i	0.55 (–4.58, 5.73)
4PLC -2PLC	0.01 [–0.22, 0.25]d	0.20 (–1.97, 2.30)				
4PLC -SPLC	0.72 [–0.25, 1.70]d	0.70 (–0.07, 1.47)	2 [0.86, 4.55]d	1.84 (0.69, 4.68)	0.15 [–2.46,2.75]d	–0.02 (–2.94, 3.23)
3PLC -2PLC	–0.58(–1.04 –0.13)i	–0.42 (–2.85, 1.98)				
3PLC -SPLC	0.13 [–0.41, 0.67]d	0.07 (–0.96, 1.10)	1.35 [0.69, 2.86]d	1.92 (0.56, 7.61)	–0.57 [–3.5 2.37]d	–0.70 (–4.39, 3.66)
2PLC -SPLC	0.71(–0.29 1.71)i	0.50 (–1.74, 2.80)				

d: DMA, direct pair-wise meta analysis; i: ITC, indirect treatment comparison meta analysis; i(4): indirect treatment comparison meta analysis via 4PLC; i(1): indirect treatment comparison meta analysis via SPLC; i(p): pooled results of indirect treatment comparison meta analysis.

*1.61 (0.30 8.80)i(4); 0.62 (0.16 2.39)i(1).

# MD [95%CI]; & MD [95%CrI]; $: RR [95%CI]; @: RR [95%CrI];

**Table 4:** Meta analysis for hospital stay and operative time.

	Hospital stay		Sensitive analysis for hospital stay		Operative time	
	DMA/ITC^#^	NMA^&^	DMA/ITC^#^	NMA^&^	DMA/ITC^#^	NMA^&^
mini-4PLC-4PLC	–0.11 [–0.31, 0.09]d	–0.13(–0.42, 0.17)	–0.11 [–0.31, 0.09]d	–0.12 (–0.40, 0.17)	5.02 [3.33, 6.70]d	5.11 (–2.64, 12.69)
mini-4PLC-3PLC	*0.33 [–0.06, 0.71]i(p)	–0.01(–0.40, 0.36)	**–0.04 [–0.46, 0.37] i(p)	–0.00 (–0.38, 0.37)	6.41 [3.21, 9.62]i(p)	3.74 (–8.28, 15.36)
mini-4PLC-2PLC	–0.16(–0.39 0.07)i	–0.13(–0.65, 0.42)	–0.16 (–0.39 0.07)i	–0.12 (–0.64, 0.41)	3.08 (–16.93 23.09)i	16.61 (–11.91, 45.73)
mini-4PLC-SPLC	–0.21 [–0.68, 0.26]d	0.06 (–0.30, 0.40)	–0.21 [–0.68, 0.26]d	0.07 (–0.28, 0.41)	1.60 [–5.29, 8.49]d	–4.99 (–15.36, 5.07)
4PLC -3PLC	0.46 [–0.10, 1.03]d	0.12 (–0.15, 0.37)	0.58 [–0.11,1.28]d	0.12 (–0.15, 0.37)	–0.13 [–3.11, 2.85]d	–1.35 (–10.78, 7.74)
4PLC -2PLC	–0.05 [–0.16, 0.06]d	0.00 (–0.46, 0.46)	–0.05 [–0.16, 0.06]d	0.00 (–0.45, 0.45)	–1.94 [–21.88, 18.00]d	11.55 (–16.07, 39.88)
4PLC -SPLC	0.16 [–0.29, 0.60]d	0.18 (–0.05, 0.40)	0.16 [–0.35,0.67]d	0.19 (–0.04, 0.40)	–16.37 [–22.75, –9.98]d	–10.05 (–17.26, –3.33)
3PLC -2PLC	–0.51 (–1.08 0.07)i	–0.11(–0.63, 0.41)	–0.63(–1.33 0.07)i	–0.12 (–0.63, 0.41)	–1.81(–21.97 18.35)i	12.87 (–16.23, 42.53)
3PLC -SPLC	0.10 [–0.06, 0.26]d	0.07 (–0.18, 0.31)	0.10 [–0.06, 0.26]d	0.07 (–0.17, 0.31)	–6.23 [–9.39, –3.07]d	–8.69 (–17.90, 0.16)
2PLC -SPLC	0.15 (0.04 0.34)i	0.18 (–0.34, 0.68)	0.21 (–0.31 0.73)i	0.19 (–0.32, 0.68)	–14.43 (–35.37 6.06)i	–21.63 (–50.74, 6.57)

d: DMA, direct pair-wise meta analysis; i: ITC, indirect treatment comparison meta analysis; i(4): indirect treatment comparison meta analysis via 4PLC; i(1): indirect treatment comparison meta analysis via SPLC; i(p): pooled results of indirect treatment comparison meta analysis.

# MD [95%CI]; & MD [95%CrI]; *:0.35 (–0.25 0.94)i(4); –0.31 (–0.81 0.19)i(1); **:0.47 (–0.23 1.19)i(4); –0.31 (–0.81 0.19)i(1); ***:4.89 (1.47 8.30)i(4); 17.97(8.58 27.36)i(1).

**Table 5:** Meta analysis for postoperative complications and cosmetic score.

	Postoperative complications			Cosmetic score		Sensitive analysis for cosmetic score	
	DMA/ITC$	NMA@	Inconsistency@	DMA/ITC#	NMA&	DMA/ITC#	NMA&
mini-4PLC-4PLC	0.61 [0.20, 1.86]d	0.31 (0.05, 1.41)	0.38 (0.06, 1.90)	1.60 [–0.05, 3.24]d	1.50 (–0.11, 3.55)	2.97 [–1.58, 7.53]d	1.60 (–0.39, 3.98)
mini-4PLC-3PLC	*0.14 [0.01, 1.94]i(p)	0.19 (0.01, 1.89)	0.09 (0.00, 1.93)	1.69 (–0.12 3.50)i	1.72 (–0.49, 4.25)	3.06 (–1.56 7.68)i	1.80 (–1.01, 5.07)
mini-4PLC-2PLC	1.56 (0.26 9.22)i	0.87 (0.03, 19.86)	1.07 (0.04, 26.67)	1.2 (–0.56 2.96)i	1.11 (–2.20, 4.78)	2.57 (–2.03 7.17)i	1.19 (–2.94, 5.68)
mini-4PLC-SPLC	0.05 [0.00, 0.98]d	0.14 (0.02, 0.77)	0.04 (0.00, 0.66)	1.01 (–0.71 2.73)i	0.61 (–1.40, 2.97)	2.47(–2.14 7.08)i	0.82 (–1.86, 3.96)
4PLC-3PLC	0.32 [0.01, 8.33]d	0.62 (0.08, 3.41)	0.25 (0.00, 3.93)	0.09 [–0.68, 0.85]d	0.20 (–1.39, 1.72)	0.09 [–0.68, 0.85]d	0.20 (–1.90, 2.35)
4PLC-2PLC	2.56 (0.63 10)d	2.72 (0.20, 50.70)	2.75 (0.20, 46.82)	–0.40 [–1.02, 0.22]d	–0.41 (–3.39, 2.65)	–0.40 [–1.02, 0.22]d	–0.38 (–4.13, 3.23)
4PLC-SPLC	0.54 (0.34 0.85)d	0.46 (0.17, 1.05)	0.49 (0.18, 1.14)	–0.59 [–1.09, –0.10]d	–0.90 (–2.14, 0.30)	–0.50 [–1.17, 0.18]d	–0.78 (–2.63, 1.17)
3PLC-2 PLC	8 (0.21 303.39)i	4.57 (0.21, 158.79)	12.67 (0.27, 2640.41)	–0.49 (–1.48 0.50)i	–0.60 (–3.84, 2.91)	–0.49(–1.48 0.50)i	–0.61 (–4.93, 3.54)
3PLC-SPLC	0.69 [0.27, 1.72]d	0.75 (0.15, 4.31)	0.75 (0.17, 4.34)	–1.13 [–0.06, –2.19]d	–1.09 (–2.44, 0.22)	–1.04 [–2.32,0.23]d	–0.98 (–2.91, 0.95)
2PLC-SPLC	0.21 (0.05 0.91)i	0.17 (0.01, 2.58)	0.06 (0.00, 2.27)	–0.19 (–0.98 0.60)i	–0.48 (–3.85, 2.64)	–0.1(–1.02 0.82)i	–0.38 (–4.34, 3.89)

d: DMA, direct pair-wise meta analysis; i: ITC, indirect treatment comparison meta analysis; i(4): indirect treatment comparison meta analysis via 4PLC; i(1): indirect treatment comparison meta analysis via SPLC; i(p): pooled results of indirect treatment comparison meta analysis.

# MD [95%CI]; & MD [95%CrI]; $: RR [95%CI]; @: RR [95%CrI];*: 0.20 (0.01 6.75)i(4); 0.07 (0.001 7.87)i(1).

### Indirect comparison (ITC) and network meta analysis (NMA)

According to the results of ITC, 3PLC was associated with shorter operative time than mini-4PLC and lower postoperative pain level than 2PLC. 2PLC was associated with fewer postoperative complication and longer hospital stay than SPLC. The NMA showed that SPLC was associated with more postoperative complications than mini-4PLC, and longer operative time than 4PLC. (Table 3, Table 4, Table 5).

### Inconsistency between DMA/ITC and NMA, heterogeneity for DMA

Node-splitting analysis (Table S1) did not detect any inconsistency among DMA, ITC and NMA except postoperative complications between mini-4PLC and SPLC. Node-splitting analysis showed that there might be inconsistency for postoperative complications (p = 0.01) among DMA, ITC and NMA. Z test did not find any inconsistency DMA/ITC and NMA (Table S2). Even so, high heterogeneity existed for most outcomes in DMA (Table S3).

### Rank probability

From the rank probability plot (Table 6), we could see that mini-4PLC has the highest level of cosmetic score, fewest postoperative complications, and lowest amount of intra-operative blood loss. 4PLC has the highest level of postoperative pain, most patients who needed additional analgesics, longest hospital stay, and lowest level of cosmetic score. SPLC has the most post-operative complications, highest amount of intra-operative blood loss, longest operative time, lowest level of postoperative pain, fewest patients who needed additional analgesics and shortest hospital stay. 2PLC has shortest operative time.

**Table 6:** Rank probability.

Drug	Pain	Additional analgesics	Complication	Blood loss	cosmetic score	sensitive analysis	Hospital stay	sensitive analysis	Operative time
SPLC	0.31	0.64	0.00	0.05	0.18	0.17	0.40	0.41	0.00
2PLC	0.24		0.45		0.19	0.21	0.16	0.16	0.76
3PLC	0.29	0.11	0.03	0.17	0.01	0.02	0.15	0.17	0.11
4PLC	0.00	0.03	0.01	0.08	0.00	0.00	0.00	0.00	0.11
mini-4PLC	0.15	0.21	0.51	0.70	0.61	0.60	0.28	0.26	0.02

## Discussion

### Summary of finding

Although DMA showed some statistical differences between different groups regarding to the outcomes we focused on, the NMA did not find any significant statistical differences except postoperative complications. However, evidence for this outcome from NMA was not consistent between DMA, ITC and NMA by node-splitting analysis. The rank probability plot suggested 4PLC might be the worst one due to the highest level of postoperative pain, most patients who needed additional analgesics, longest hospital stay, and lowest level of cosmetic score. The best one might be mini-4PLC because of highest level of cosmetic score, fewest postoperative complications, and lowest amount of intra-operative blood loss or SPLC because of lowest level of postoperative pain, fewest patients who needed additional analgesics and shortest hospital stay. However, SPLC has most post-operative complications and highest amount of intra-operative blood loss.

For postoperative pain at the first day, significant differences existed between 3PLC and 4PLC (DMA), 3PLC and 2PLC (ITC). The rank probability showed SPLC might be the best in reducing the first day postoperative pain, and 4PLC might be the worst. Although the inconsistency between DMA or ITC and NMA could not be detective by node-splitting analysis and Z test, the heterogeneity among included studies for direct evidence existed. That might be because of different anesthetics used before surgery and anesthetic prophylaxes after surgery. Due to this point, we did not calculate the amount of anesthetics consumption; we calculated the number of patients who required additional analgesics. And that was why we used the postoperative first day pain level that was measured using VAS at the first postoperative day. This is consistent with the results of the number of patients who required additional analgesics. The rank probability showed that patients in 4PLC group used the most additional analgesics and patients in SPLC group used the fewest additional analgesics, although no significant differences were found in DMA, ITC and NMA.

For postoperative complication, significant differences existed between SPLC and mini-4PLC (DMA), SPLC and 4PLC (DMA and NMA), SPLC and 2PLC (ITC). Rank probability showed that mini-4PLC was associated with fewest postoperative complications, and SPLC was associated with most postoperative complications. Among the included studies, 18 studies reported postoperative complications for SPLC with a median rate of 6.46% (0%–35.71%), one studies reported postoperative complications for 2PLC (5.17%), five studies reported postoperative complications for 3PLC with a median rate of 3.33% (1.96%–9.43%), 19 studies reported postoperative complications for 4PLC with a median rate of 6.17% (0%–48.28%), five studies reported postoperative complications for mini-4PLC with a median rate of 2.50% (0%–8.57%). However, node-splitting analysis showed there were inconsistencies between mini-4PLC and SPLC, 4PLC and 3PLC, although Z test did not find any inconsistency between DMA/ITC and NMA evidence and there were not high heterogeneity for the direct evidence. So inconsistence model in ADDIS software was used, but similar results were found.

For cosmetic scores, statistical significances existed between SPLC and 3PLC, SPLC and 4PLC. And the rank probability showed that mini-4PLC has the best cosmetic scores, and 4PLC has the worst cosmetic scores. Although no any inconsistency existed, high heterogeneity was common among direct comparisons. The high heterogeneity might be because of different measurements for cosmetic score. Some studies used a five-point scale [20], [33], [47], some studies used a ten-point scale [31], [44], [49], [56], [58], some studies used other scale, such as 24 points [40], 40 points [42]. Sensitive analysis was conducted to analyze the cosmetic scores among studies who used ten-point scale. The results for DMA, ITC and NMA did not show any statistical differences. And the rank probability for sensitive analysis was consistent with the previous probability.

For hospital stay, DMA and NMA did not show any significant differences; only ITC showed that 2PLC was associated with longer hospital stay than SPLC. And the rank probability showed SPLC was associated with shortest hospital stay, and 4PLC was associated with longest hospital stay. Due to some studies used hours to measure the length of hospital stay, we conduct sensitive analysis. Sensitive analysis of DMA, ITC and NMA showed no differences among any two comparisons. And the rank probability of sensitive analysis was consistent with the previous one. As LC has a faster recovery, many hospitals conducted day-surgery rather than overnight stay surgery. And culture and hospital types could also affect the length of hospital stay. And these factors might be the reasons for the heterogeneity of the direct evidence.

Two operative outcomes, operative time and intra-operative blood loss, were evaluated. Significances existed between SPLC and 4PLC, SPLC and 3PLC, mini-4PLC and 4PLC (DMA), mini-4PLC and 3PLC (ITC), SPLC and 4PLC (NMA) for operative time. The rank probability showed that SPLC was associated with the longest operative time, and 2PLC was associated with the shortest operative time. For intra-operative blood loss, no significant differences were found in DMA, ITC and NMA.

There were several systematic reviews [1], [11], [13], [15], [59]–[62] published in 2013. And the results of our DMA were consistent with their results. Similar to these meta analysis, high heterogeneity was common, although we strictly restricted studies to those which used the same measurement at the same time, for example, postoperative pain using VAS at the first day. We also conducted sensitive analysis by excluding studies which used different measurement units, but results did not change for DMA. ITC was also conducted when there was no DMA evidence. Although no inconsistencies were found between DMA/ITC and NMA using Z test, node-splitting analysis showed there were not any inconsistencies among DMA, ITC and NMA except postoperative complications. Although we used inconsistency model to analyze the data, the results and conclusions did not change.

### Strengths and limitations

This is the first ITC and NMA which compared different kinds of LC. We also calculated the inconsistency using node-splitting analysis and Z test. Inconsistency model and sensitive analysis were used to test the stability of the results, and the results did not change for DMA and NMA. However, our NMA has its own limitations: first, our NMA only included studies which specified how many ports they used during their surgery. For those studies that it is hard to judge whether 4PLC or 3PLC, we excluded them. For example, study conducted by Vilallonga [63] did not specify what their standard LC is, so we excluded it. Second: we did not include quasi-randomized studies. For example, we excluded two studies [64], [65] as they used quasi-randomized study design. We included lots of studies (30/43) which just mentioned randomization, but they did not report the detail of the randomization. Due to the high risk of bias in most of the studies, the results of our DMA, ITC and NMA might be biased. Third: the heterogeneity for DMA is high. It was said that heterogeneity between the sets of studies that contribute direct comparisons to an indirect comparison or a network meta-analysis would indicate a lack of similarity [66]. We checked the clinical and methodological similarity among all included studies, and then we found indeed there were some differences among all included studies, such as different analgesics used before and after surgery, different instruments during the surgery, studies from different countries, and some other variances for the LC. Even so, inconsistency was not found for most outcomes, except postoperative complications. However, the inconsistency model did not change the results. Fourth: there were many factors that might affect length of hospital stay, such as culture differences and hospital types; however, we did not conduct subgroup analysis due to limited data.

### Implications to future research and practice

Most included studies did not mention the details of randomization and concealed allocation, nearly all of them were of small sample size. In the future randomized controlled studies of big sample size should be well conducted and adequately reported. For outcomes, such as postoperative pain, hospital stay should be measured using international standards, such as VAS for pain, day for hospital stay. Regarding to cosmetic scores, too many scales were used in the primary studies, which scale will be better to measure the cosmetic satisfaction? This needs a comparative study to test the validity of different scales. Based on our NMA, we could see that 4PLC might be the worst, but it is hard to decide which one is the best, as few studies compared SPLC with mini-4PLC. The rank probability showed that either SPLC or mini-4PLC will be the best, although SPLC has the most post-operative complications, highest amount of intra-operative blood loss, and longest operative time. As a result, future more studies were needed to compare SPLC with mini-4PLC.

Based on the rank probability, we should make sure to let patients know that SPLC was associated with lowest postoperative pain, most postoperative complications, and longest hospital stay, mini-4PLC was associated with high level cosmetic score and fewest complications. For surgeons, when conducting SPLC, please pay attention to the intra-operative blood loss and postoperative complications.

## Supporting Information

Table S1 **Node-splitting analysis.**
(DOC)

Table S2 **Z test for inconsistency.**
(DOC)

Table S3 **I^2^ test for heterogeneity.**
(DOC)

Checklist S1 **PRISMA checklist.**
(DOC)
