# Supplementary material for: The Efficacy and Safety of Different Kinds of Laparoscopic Cholecystectomy: A Network Meta Analysis of 43 Randomized Controlled Trials
Source: PLoS One. 2014 Feb 28;9(2):e90313. doi: 10.1371/journal.pone.0090313 (PMC3938681; doi:10.1371/journal.pone.0090313)
Supplement: Table S1 — Node-splitting analysis. (DOC) [file pone.0090313.s001.doc]

Supplement table 1 Node-splitting analysis

| NMA  (P) | Postoperative pain | Additional analgesics | Postoperative complications | Cosmetic score | Sensitive analysis | Hospital stay | Sensitive analysis | Operative time |
| --- | --- | --- | --- | --- | --- | --- | --- | --- |
| mini-4PLC-1 PLC |  | 0.58 | 0.01 |  |  | 0.74 | 0.76 | 0.60 |
| 4PLC -3PLC | 0.98 | 0.72 | 0.04 | 0.90 | 0.95 | 0.47 | 0.44 | 0.57 |
| 4PLC -SPLC | 0.95 |  |  | 0.66 | 0.67 | 0.60 | 0.58 | 0.46 |
| 3PLC -SPLC | 0.95 |  |  | 0.64 | 0.67 | 0.42 | 0.40 | 0.59 |
