# Supplementary material for: The Efficacy and Safety of Different Kinds of Laparoscopic Cholecystectomy: A Network Meta Analysis of 43 Randomized Controlled Trials
Source: PLoS One. 2014 Feb 28;9(2):e90313. doi: 10.1371/journal.pone.0090313 (PMC3938681; doi:10.1371/journal.pone.0090313)
Supplement: Table S2 — Z test for inconsistency. (DOC) [file pone.0090313.s002.doc]

Supplement table 2 Z test for inconsistency

| NMA  Z | Postoperative pain | Additional analgesics | Postoperative complications | Blood loss | Cosmetic score | Sensitive analysis for cosmetic score | Hospital stay | Sensitive analysis for hospital stay | Operative time |
| --- | --- | --- | --- | --- | --- | --- | --- | --- | --- |
| mini-4PLC-4PLC | -0.06 | -0.27 | -0.66 | -0.12 | -0.08 | -0.53 | -0.11 | -0.06 | 0.02 |
| mini-4PLC-3PLC | -0.09 | -0.13 | 0.16 | -0.10 | 0.02 | -0.45 | -1.23 | 0.14 | -0.43 |
| mini-4PLC-2PLC | 0.08 |  | -0.31 |  | -0.05 | -0.43 | 0.10 | 0.14 | 1.01 |
| mini-4PLC-SPLC | -0.19 | 0.67 | 0.41 | -0.11 | -0.28 | -0.59 | 0.90 | 0.94 | -1.05 |
| 4PLC -3PLC | -0.05 | -0.51 | 0.34 | -0.05 | 0.12 | 0.10 | -1.07 | -1.22 | -0.25 |
| 4PLC -2PLC | 0.15 |  | 0.04 |  | -0.03 | 0.01 | 0.21 | 0.21 | 0.77 |
| 4PLC -SPLC | -0.23 | -0.13 | -0.31 | -0.08 | -0.45 | -0.27 | 0.08 | 0.11 | 1.31 |
| 3PLC -2PLC | 0.17 |  | -0.22 |  | -0.06 | -0.05 | 1.01 | 1.15 | 0.81 |
| 3PLC -SPLC | -0.19 | 0.46 | 0.09 | -0.05 | 0.05 | 0.05 | -0.20 | -0.20 | -0.71 |
| 2PLC -SPLC | -0.25 |  | -0.13 |  | -0.17 | -0.13 | 0.11 | -0.05 | -0.40 |
