# Supplementary material for: The Efficacy and Safety of Different Kinds of Laparoscopic Cholecystectomy: A Network Meta Analysis of 43 Randomized Controlled Trials
Source: PLoS One. 2014 Feb 28;9(2):e90313. doi: 10.1371/journal.pone.0090313 (PMC3938681; doi:10.1371/journal.pone.0090313)
Supplement: Table S3 — I2 test for heterogeneity. (DOC) [file pone.0090313.s003.doc]

Supplement table 3 I^2^ test for heterogeneity

| DMA  I² | Postoperative pain | Additional analgesics | Postoperative complications | Blood loss | Cosmetic score | Sensitive analysis for cosmetic score | Hospital stay | Sensitive analysis for hospital stay | Operative time |
| --- | --- | --- | --- | --- | --- | --- | --- | --- | --- |
| mini-4PLC-4PLC | 82% | 0% | 0% |  | 93% | 94% | 17% | 17% | 9% |
| 4PLC -3PLC | 0% |  |  |  | 71% | 71% | 90% | 92% | 26% |
| 4PLC -2PLC | 0% |  |  |  |  |  | 46% | 46% | 72% |
| 4PLC -SPLC | 95% | 49% | 30% | 0% | 91% | 91% | 88% | 89% | 90% |
| 3PLC -SPLC | 68% | 14% | 0% | 81% | 97% | 98% | 65% | 65% | 59% |
